# What drives China’s healthcare expenditure? A theoretical and empirical study of determinants and trends

**DOI:** 10.3389/fpubh.2024.1445912

**Published:** 2024-09-04

**Authors:** Zhilin Ge, Jinfeng Cai, Jingbo Hu

**Affiliations:** ^1^School of Finance, Jilin University of Finance and Economics, Changchun, China; ^2^Business School of Changchun Guanghua University, Changchun, China

**Keywords:** healthcare expenditure, impact factor, SV-TVP-FAVAR model, economic, time-varying

## Abstract

As economic development advances, there is an increasing focus on improving health conditions, making healthcare expenditure a critical issue worldwide. In China, healthcare spending has shown a marked upward trend, highlighting the importance of understanding its underlying determinants to guide effective policy-making. This study introduces the application of an SV-TVP-FAVAR model to examine the drivers of healthcare expenditure in China from 2007 to 2022. The analysis reveals that economic factors, demographic composition, and policy interventions significantly influence healthcare spending dynamics. Economic growth is strongly linked to increased healthcare expenditure, with economic factors having a particularly pronounced impact during periods of prosperity. Although an aging population drives greater demand for healthcare, the growth rate of healthcare spending has not kept pace with demographic aging, especially following China’s economic slowdown. Policy variables present a dual-edged impact: while increased fiscal outlays contribute to budget deficits, limiting the fiscal space for healthcare investment, government emphasis on scientific and technological progress tends to enhance healthcare spending, indicating a synergistic relationship between these areas. Furthermore, the study identifies a prolonged impact of the COVID-19 pandemic on healthcare expenditure, which continues to interact with other driving factors over an extended period. The empirical findings from this research provide crucial evidence to support the development of informed healthcare policies.

## 1 Introduction

**Figure 1:**
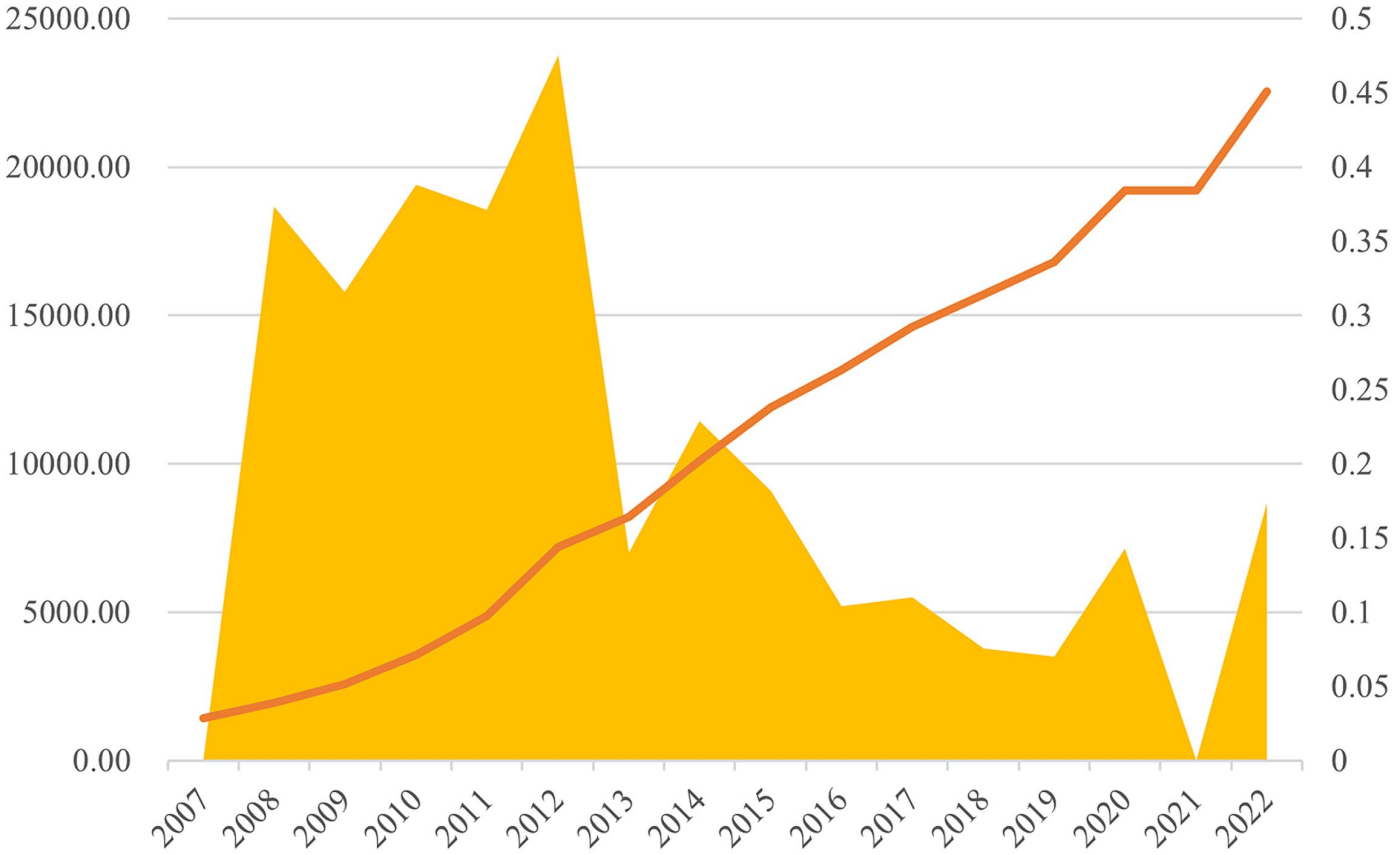
Annual total value of China’s healthcare expenditure (unit: 100 million yuan) and annual growth rate.

The nexus between economic development, evolving health consciousness, and the disruptive impact of pandemics has underscored an escalating appreciation for the intrinsic value of health, thereby amplifying efforts aimed at ameliorating health conditions. Healthcare expenditure epitomizes a nation’s commitment to the medical and health sector from a societal standpoint (1, 2), correlating closely with health outcomes such as diminished mortality rates and prolonged life expectancy. Notably, China has experienced a marked surge in healthcare expenditure in recent years. Illustrated in Figure 1, the allocation of public financial resources to healthcare in China surged from 141.885 billion yuan in 2007 to 2,254.200 billion yuan in 2023. Over this period, the growth rate of healthcare expenditure in China peaked at 47.51%, stabilizing below 10% after 2015. The outbreak of the COVID-19 pandemic in late 2019 had profound global repercussions (3, 4). In response to the pandemic’s negative impacts, China’s healthcare expenditure growth surged to 14.31%. The expenditure levels in 2021 remained consistent with those of 2020, and in 2022, the growth rate further increased to 17.38%, significantly outpacing GDP growth during the same period. This trend underscores the Chinese government’s heightened focus on the healthcare sector, robust health measures are essential at every level (5).

China’s fiscal healthcare expenditure primarily focuses on the medical service delivery system, public health service system, and basic healthcare insurance system. In 2009, a new phase of healthcare reform began, with healthcare expenditure surpassing 250 billion yuan, signaling a significant resurgence in government financing in the healthcare sector. This period saw not only rapid expansion in the scale of fiscal healthcare spending but also a shift in investment priorities away from the previously hospital-centric approach. The new healthcare reform plan emphasizes two key aspects of fiscal healthcare expenditure allocation: first, the government’s financial support to enhance healthcare service capacity, directly addressing the issue of “access to care”; second, multi-level fiscal backing aimed at developing a multi-tiered healthcare insurance system to mitigate the challenge of “high healthcare costs.” Despite ongoing challenges in China’s healthcare insurance system, the multi-tiered framework, primarily centered on basic healthcare insurance, is steadily gaining strength. Coverage under employee medical insurance, urban resident medical insurance, and the new rural cooperative medical scheme now exceeds 1.3 billion individuals, achieving a coverage rate of over 95% and establishing it as the world’s most extensive basic healthcare security network.

Previous research has primarily focused on the impact of healthcare expenditure on areas such as population health, life expectancy, and multidimensional poverty. The advent of digital technology (6), however, has brought new perspectives to this field. In particular, the relationship between healthcare expenditure and population health has received significant scholarly attention. Although most existing research suggests strong institutional support for healthcare services leads to better population health outcomes, some studies argue that the relationship between healthcare spending and health improvements remains unclear (7–9), positing potential oversight regarding the long-term ramifications of healthcare expenditure. Despite the recent surge in China’s healthcare expenditure, it remains lower than that of Western developed countries. However, unchecked rapid growth in healthcare spending could intensify economic burdens, potentially surpassing socioeconomic capacity and posing significant challenges to the sustainability of healthcare financing and the long-term development of healthcare infrastructure. Understanding the determinants of healthcare expenditure is therefore crucial for policymakers, as it enables them to grasp the underlying drivers of expenditure fluctuations and assess the varying impacts of different factors, ultimately supporting the formulation of sound and sustainable healthcare policies.

Current research on the determinants of healthcare expenditure spans diverse areas, including demographics, economics, disease epidemiology, education, environmental quality, and atmospheric conditions. These studies have provided valuable insights from various perspectives. However, disparities in healthcare insurance systems, economic development, population demographics, and cultural contexts across countries lead to variations in the factors influencing healthcare expenditure. As a result, there is no standardized approach for assessing the impact of these determinants across different national contexts, and subjective biases may affect the analysis.

Moreover, previous studies have predominantly employed static or relatively static methodologies, such as instrumental variable techniques, generalized autoregressive models, and logistic regression, to investigate the drivers of healthcare expenditure. Yet, healthcare expenditure is subject to fluctuations due to exogenous shocks like health crises or policy reforms. The impact of different determinants on healthcare expenditure is also context-dependent. Therefore, relying on static or relatively static methods may introduce biases, highlighting the need for more dynamic approaches in analyzing healthcare expenditure determinants.

In this vein, this study espouses the SV-TVP-FAVAR model, amalgamating diverse determinants into the analytical framework, encompassing a myriad of variables intrinsic to China’s healthcare expenditure trajectory. Leveraging a factor-augmented approach, it surmounts the limitations inherent in antecedent methodological paradigms concerning extensive data computations. Moreover, this study adopts a dynamic perspective in probing the determinants of healthcare expenditure, spotlighting the disparate impacts engendered by analogous determinant shocks on healthcare expenditure across distinct temporal epochs. This augurs well for judiciously modulating healthcare expenditure levels, thereby ensuring equitable provisioning of fundamental medical and public health amenities to both urban and rural denizens.

The subsequent sections of this manuscript are structured as follows: Section 2 delineates the methodology employed in model construction, while Section 3 furnishes an exhaustive exposition of the pertinent variables. Section 4 undertakes dynamic simulations to elucidate the driving forces behind healthcare expenditure in China. Finally, the concluding section encapsulates the entirety of the paper and proffers policy implications.

## 2 Methodology

### 2.1 Model construction

The factors influencing healthcare expenditure (HCE) are often multifaceted, and past research on identifying the drivers of HCE has faced challenges, particularly in accurately isolating the determinants of HCE from a vast array of influencing factors. Additionally, the impact of the same factors on HCE may vary under different economic conditions, necessitating a dynamic perspective when analyzing the drivers of HCE. To address the limitations of previous studies, this paper employs the SV-TVP-FAVAR model to analyze the drivers of China’s HCE. This model, on the one hand, incorporates factor-augmented approaches, enabling the processing of large volumes of information and mitigating the “curse of dimensionality” faced by classical models. On the other hand, its time-varying nature allows for a dynamic analysis of HCE drivers under different economic conditions in recent years, thereby avoiding potential biases from overlooking the time-varying characteristics of HCE drivers in previous studies. This approach is essential for accurately examining the factors influencing HCE.

In order to study the determinants of HCE, we first start with a classic VAR model as shown in Equation 1:


(1) $$Ay_{t}=\beta_{1}y_{t-1}+\cdots +\beta_{s}y_{t-s}+u_{t},t=s+1,\ldots ,n$$

Where $y_{t}$ is a l×1 vector of observed variables, representing China’s healthcare expenditure (HCE). *A* is a l×l vector coefficient matrix, $\beta_{1},\beta_{2},\cdots \beta_{s}$ is also l×l dimensions. $u_{t}$ is a disturbance item, which represents an exogenous impact. According to the setting of the classic VAR model (10), *A* should be a lower triangular matrix. From this, the classic VAR model can be written as follows (11, 12) as shown in Equation 2:


(2) $$y_{t}=B_{1}y_{t-1}+\cdots +B_{s}y_{t-s}+A^{-1}\Sigma \varepsilon_{t},\varepsilon_{t}∼N\left(0,I_{k}\right)$$

Where $B_{i}=A^{-1}\beta_{i}$ , for i=1,⋯,s . It is evident that the classic VAR model, being a constant-parameter model, is unable to address time-varying issues and typically handles only a limited number of variables. Therefore, it is essential to extend the classic VAR model to better accommodate these complexities.

Where the elements in the matrix Σ are the standard deviations of structural shocks. Define $X_{t}=I_{k}⊗\left(y_{t-1}^{\prime },\cdots y_{t-k}^{\prime }\right)$ , the model can be rearranged as (13, 14) as shown in Equation 3:


(3) $$y_{t}=X_{t}\gamma +A_{t}^{-1}\Sigma_{t}\varepsilon_{t}$$

γ is a $k^{2}s\times 1$ dimension vector composed of $B_{i}^{'}$ . Further, we can define every parameter in the VAR model after finishing as time-varying, then there are Equation 4:


(4) $$y_{t}=X_{t}\gamma_{t}+A^{-1}\Sigma_{t}\varepsilon_{t},t=s+1,\cdots ,n$$

Coefficient matrix $\gamma_{t}$ , parameters $A_{t}$ and $\Sigma_{t}$ are all time-varying, Let at be a stacked vector of the lower-triangular elements in $A_{t}$ , and have $h_{jt}=\log\sigma_{jt}^{2}$ , j=1,⋯,k , t=s+1,⋯n . All relevant parameters should follow the innovative random walk form as shown in Equation 5:


(5) $$\begin{array}{l} \gamma_{t+1}=\gamma_{t}+u_{\gamma t},\\ a_{t+1}=a_{t}+u_{\mathrm{at}},\\ h_{t+1}=h_{t}+u_{ht}\text{,} \end{array}\left(\begin{array}{c} \varepsilon_{t}\\ u_{\gamma t}\\ u_{\mathrm{at}}\\ u_{ht} \end{array}\right)N\left(0,\left(\begin{array}{cccc} I & O & O & O\\ O & \Sigma_{\gamma } & O & O\\ O & O & \Sigma_{a} & O\\ O & O & O & \Sigma_{h} \end{array}\right)\right)$$

Where $\gamma_{s+1}∼N\left(\mu_{\gamma 0},\Sigma_{\gamma 0}\right)$ , $a_{s+1}∼N\left(\mu_{a0},\Sigma_{a0}\right)$ and $h_{s+1}∼N\left(\mu_{h0},\Sigma_{h0}\right)$ , t=s+1,⋯,n . $\mu_{\gamma 0}$ , $\mu_{a0}$ and $\mu_{h0}$ all come from the OLS estimation of the pre-sample data, $\Sigma_{\gamma 0}$ , $\Sigma_{a0}$ and $\Sigma_{h0}$ all are diagonal matrices. In order to further enable the model to deal with high-dimensional problems, a factor extraction equation is constructed as Equation 6:


(6) $$X_{t}=\Psi^{uv}uv_{t}+\Psi^{v}v_{t}+e_{t}$$

$X_{t}$ is $\left(N\times 1\right)$ dimensional information set, cover as many factors as possible that affect China’s HCE. $uv_{t}$ and $v_{t}$ the unobservable part and the observable part, respectively. $\Psi^{uv}$ and $\Psi^{v}$ express the factor loading matrix of $\left(N\times L\right)$ dimension and $\left(N\times M\right)$ dimension, N≫L+M , $\varepsilon_{t}\sim N\left(0,\Omega_{t}\right)$ . The extended model is capable of handling high-dimensional data, with all parameters being time-varying, making it more suitable for studying the drivers of China’s healthcare expenditure (HCE).

### 2.2 Variable selection and data description

As mentioned earlier, the driving factors for healthcare expenditure vary across countries. Therefore, this study selects the potential influencing factors based on the characteristics of China. Compared to other studies, this paper adopts a factor-enhanced approach, which allows for the consideration of more influencing factors and provides a clearer description of the driving factors of healthcare expenditure. Table 1 presents the selected driving factors, which are obtained from the Wind database. Considering that the economy has always been an important factor influencing healthcare expenditure (15), this study selects China’s Consumer Price Index (CPI) and Gross Domestic Product (GDP) as the driving factors for healthcare expenditure in China. CPI measures the degree of inflation, while GDP reflects the overall economic output of a country. Strengthened economic power and increased consumer capacity will promote higher expectations for health among residents. In addition to the economy, population structure is also an important influencing factor for healthcare expenditure (16). As the degree of population aging deepens, healthcare systems face more prominent challenges. Therefore, this study selects the proportion of the population aged 65 and above as a relevant indicator for measuring aging. At the same time, Labor Force (LAB) is selected to measure the actual economic active population in China, providing another perspective to verify the driving effect of population structure-related variables on healthcare expenditure. Policy factors can also significantly influence healthcare expenditure (17). The government can influence a country’s healthcare sector in various ways and plays a leading role in China’s healthcare system. The government’s investment in health reflects its attention to health and people’s livelihood issues. Therefore, this study selects the variables Rev. and STE as proxies for policy factors.

**Table 1:** Descriptive statistics of the main variables.

Var.	Var. def.	Sam. per.	Mean	Max.	Min.	Std. err.	Median
HCE	Health care expenditure in GDP (%)	2007Q1–2022Q4	0.031	0.084	0.007	0.003	0.023
CPI	China’s consumer price index: month-on-year (%)	2007Q1–2022Q4	7.761	24.100	−4.600	0.710	6.607
GDP	China’s GDP quarterly growth rate (%)	2007Q1–2022Q4	7.849	18.700	−6.900	0.478	7.500
Dem	Demographics as measured by proportion of 65 years old and above in China’s total population (%)	2007Q1–2022Q4	10.580	14.900	7.950	0.254	10.150
LAB	Labor force participation as measured by logarithm of China’s economically active population	2007Q1–2022Q4	11.270	11.291	9.374	0.057	10.431
Rev	Logarithm of China’s fiscal revenue	2007Q1–2022Q4	10.282	11.002	9.374	0.057	10.431
STE	Logarithm of China’s science and technology fiscal expenditures	2007Q1–2022Q4	6.940	8.284	4.810	0.100	6.977

Sam. Per. represents the sample period; Var. = variable; Max. = maximum; Min. = minimum; Std. Err. = standard error.

**Figure 2:**
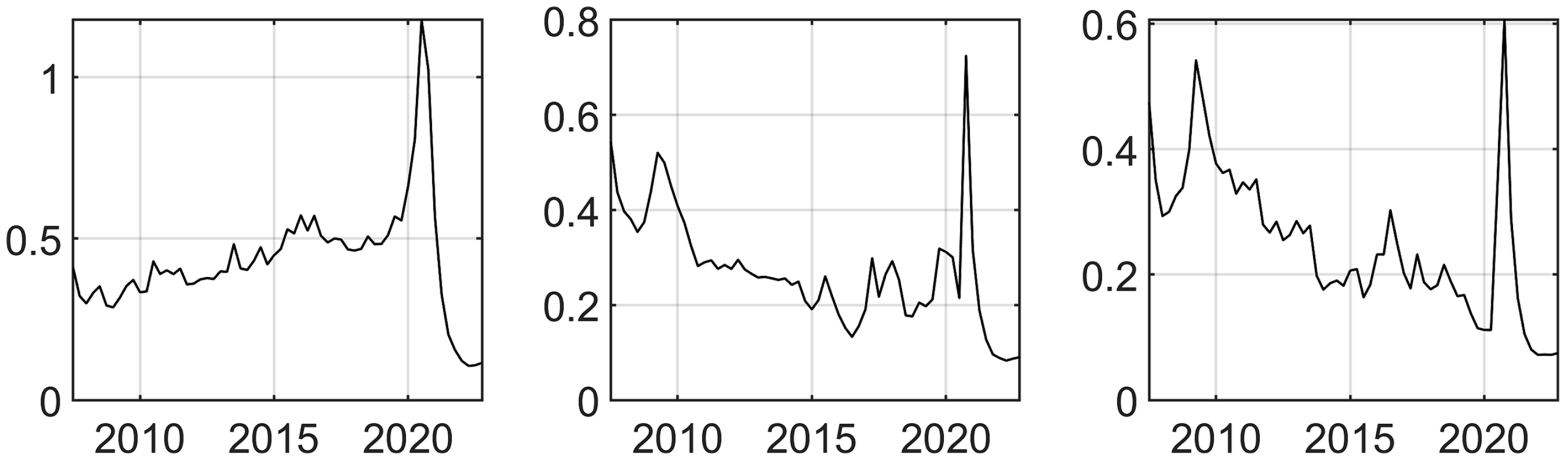
The trend of the posterior mean of the extracted common factors.

To mitigate the risk of overlooking pertinent variables influencing healthcare expenditure, supplementary factors associated with the aforementioned aspects are integrated into a cohesive analytical framework as background datasets. To circumvent the “curse of dimensionality” stemming from an excessive array of variables, a factor-augmented methodology is employed to manage the related variables. Through this approach, three latent factors are derived, and the posterior mean trends of these factors are depicted graphically in Figure 2. It is discernible that during periods marked by significant economic or policy shifts, the posterior mean trends of the related factors exhibit conspicuous fluctuations. The extracted latent factors serve as proxies capturing the predominant information embedded within the original dataset.

## 3 Empirical results

### 3.1 An empirical test of the driving factors of HCE in China

**Figure 3:**
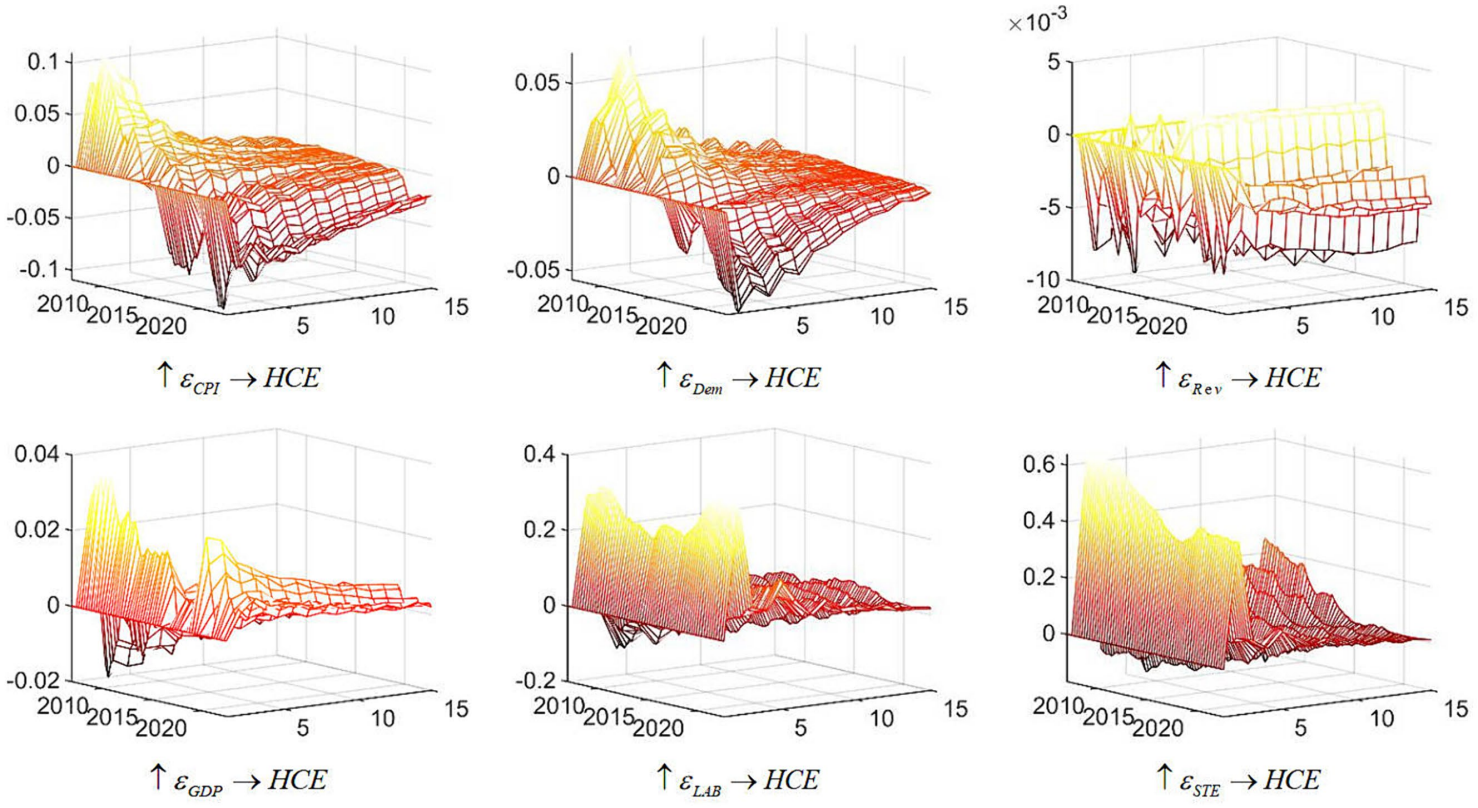
Three-dimensional impulse response of China’s health care expenditure. Note: Each subfigure with the title of “ $↑\varepsilon_{X}\to Y$ ” demonstrates the response of variable Y to an orthogonalized positive shock to variable X. One period in the figure denotes one season.

Figure 3 elucidates the prospective influence of driving factors on healthcare expenditure in China. Within the three-dimensional depiction, the X-axis denotes the onset time of the shock, the Y-axis delineates the subsequent response period following the shock, each unit representing a quarter, and the Z-axis portrays the intensity of response. The plane defined by XOZ embodies the temporal dimension, signifying the extent of healthcare expenditure response in China triggered by driving factors across distinct temporal epochs. Conversely, the plane delineated by YOZ represents the response dimension, elucidating the reaction of healthcare expenditure in China instigated by driving factors at specific temporal junctures.

It is evident that various driving factors significantly affect healthcare expenditure in China, with their impacts evolving over time. Prior to 2015, a positive unit increase in the Consumer Price Index (CPI) led to an increase in healthcare expenditure. However, post-2015, this effect became increasingly negative. Since 2020, the response of healthcare expenditure to CPI has shown signs of structural change, characterized by a notably slower convergence rate. Regarding Gross Domestic Product (GDP), an overall increase in GDP generally promotes healthcare expenditure. Nonetheless, the influence of GDP on healthcare expenditure has diminished annually until the COVID-19 pandemic, which triggered a resurgence in GDP’s impact on healthcare expenditure, accompanied by a prolonged response duration.

The impact of the aging index on healthcare expenditure in China reflects a pattern similar to that of CPI, with a pronounced initial effect that diminishes over time, often turning negative. Notably, the influence of the aging index on healthcare expenditure extends beyond the COVID-19 pandemic. Enhanced labor force participation is associated with increased healthcare expenditure, displaying a U-shaped pattern: an initial decline followed by subsequent growth. The response to a positive shock in labor force participation converges relatively quickly. Regarding policy factors, an increase in fiscal revenue leads to a negative reaction in healthcare expenditure, with gradual convergence and structural effects. Conversely, a positive shock in science and technology fiscal expenditures results in an increase in healthcare expenditure, characterized by a slow initial decline, followed by an increase and a relatively swift convergence.

The tridimensional analysis of driving factors reveals that labor force participation and fiscal expenditures on science and technology significantly impact healthcare expenditure dynamics in China. Among economic determinants, Gross Domestic Product (GDP) reflects overall economic output, while the Consumer Price Index (CPI) measures inflationary pressures. In periods of strong economic growth, increased residents’ incomes and greater government capacity to invest in public health lead to higher healthcare expenditure. Furthermore, robust economic conditions enhance consumer spending, causing a positive CPI shock to stimulate healthcare expenditure. However, as the economy stabilizes, the impact of CPI on healthcare expenditure diminishes.

Additionally, with rising life expectancy and declining birth and mortality rates, the proportion of the older adult population continues to grow, intensifying the challenges associated with healthcare expenditure. The global aging trend further exacerbates these challenges in China, as the expanding older adult demographic increases societal burdens and the demand for healthcare services, thereby driving up healthcare expenditure.

Policy dynamics exhibit a complex influence on healthcare expenditure. The government plays a crucial role in shaping public health and influencing the healthcare system. A positive shock in fiscal revenue results in a negative response in healthcare expenditure due to fund reallocation, where increased spending in other sectors reduces available resources for public health. Conversely, higher fiscal allocations to science and technology reflect a period of emphasis on innovation, which often accompanies increased investment in public health. This is evidenced by the significant impact of China’s science and technology expenditures on healthcare expenditure.

### 3.2 Robustness test

**Figure 4:**
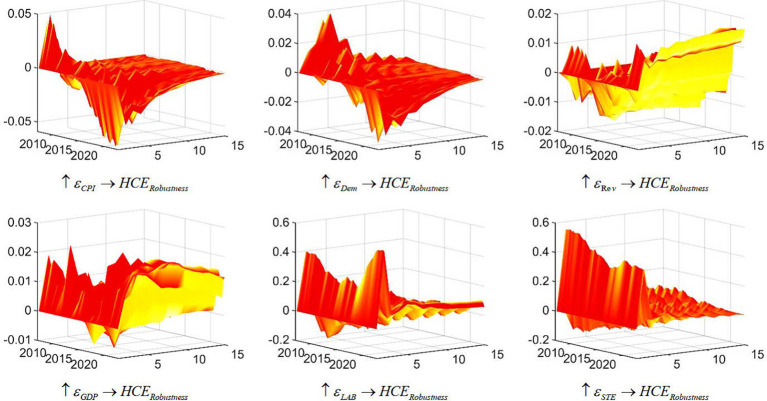
Three-dimensional impulse response of China’s health care expenditure (robustness test). Each subfigure with the title of “ $↑\varepsilon_{X}\to Y$ ” demonstrates the response of variable Y to an orthogonalized positive shock to variable X. One period in the figure denotes one season.

To ensure the resilience and reliability of the empirical findings, a robustness test is conducted to assess the impact of driving factors on healthcare expenditure in China. The primary variable utilized to gauge healthcare expenditure in China is denoted as “Health care expenditure in GDP,” represented by the variable “The personnel expenditure of public health institutions and the sum of public health service expenditures” in the Wind database^1^. In this examination, the variable “the proportion of healthcare expenditure in GDP (%)” is substituted, and the ramifications of diverse driving factors on healthcare expenditure in China are simulated. The results, depicted in the Figure 4, affirm that the substitution of the variable does not alter the principal conclusions of this study, thus corroborating the steadfastness of the research findings.

## 4 Discussion

This study contributes to the ongoing debate about the determinants of healthcare expenditure in China, particularly in light of varying perspectives in the literature. While existing research often highlights the positive impact of strong institutional support on population health outcomes, there are contrasting views that question the direct relationship between healthcare expenditure and improvements in health. Our findings align with the nuanced understanding that healthcare expenditure alone does not unequivocally lead to better health outcomes, emphasizing the role of economic, demographic, and policy factors in shaping these dynamics.

Economic growth is a key driver of healthcare spending, especially during periods of prosperity, yet the recent economic deceleration has revealed a disconnect between the needs of an aging population and actual healthcare expenditure. This misalignment suggests that healthcare expenditure must be strategically managed to address the evolving demographic pressures. Furthermore, policy interventions exhibit a dual effect: while increased fiscal spending has strained public budgets, investments in science and technology have positively influenced healthcare expenditure. The lingering effects of the COVID-19 pandemic further complicate the relationship between spending and health outcomes, highlighting the need for a more nuanced and adaptable approach to healthcare policy.

In summary, this study underscores the complexity of the relationship between healthcare expenditure and population health, advocating for a balanced and dynamic approach to policymaking that considers the multifaceted influences of economic, demographic, and policy variables.

## 5 Conclusion

Based on our analysis, several key conclusions emerge. First, economic development closely influences healthcare expenditure, with the Chinese government’s investments significantly easing population burdens and showing heightened sensitivity during economic upswings. Second, demographic shifts, particularly an aging population, significantly impact healthcare spending. Despite rising demand, expenditure growth has not aligned with aging trends due to economic slowdowns, highlighting the need for targeted government attention. Third, policy interventions have a dual effect: increased fiscal spending can strain budgets, while investment in science and technology often boosts healthcare funding, demonstrating a beneficial interplay. Lastly, the extended impact of the COVID-19 pandemic on healthcare expenditure underscores a more complex, fragile system. The SV-TVP-FAVAR model’s capacity to address time-varying and high-dimensional data is crucial for informing effective policy decisions.

Policymakers should adopt flexible fiscal strategies that balance healthcare spending with economic conditions and address demographic challenges through targeted investments. Emphasizing technological advancements while managing fiscal constraints is crucial. Additionally, robust contingency plans for health crises are essential.

Future studies should use dynamic models to capture time-varying impacts on healthcare expenditure, conduct comparative international research, explore the role of technological innovations, and perform longitudinal analyses to understand long-term effects.

## Data Availability

Publicly available datasets were analyzed in this study. This data can be found here: the datasets we collected come from Wind, and it has been indicated in the article.

## Footnotes

^1^The specialized public health service network in China includes professional service institutions such as disease prevention and control, health education, and maternal and child health care, as well as grassroots medical and health institutions with integrated medical and preventive functions. In urban areas, these are community health service institutions, while in rural areas, they are township health centers and village clinics, collectively forming an interconnected public health service system. Therefore, in this study, the sum of the financial subsidies received by specialized public health institutions and grassroots medical and health institutions is used as a proxy variable for public health expenditure.
